# Feasibility and Acceptability of an AI-Driven Conversational Platform for Structured Autism History Taking and Referral Support: Mixed Methods Proof-of-Concept Study

**DOI:** 10.2196/100237

**Published:** 2026-08-27

**Authors:** Shabnam Sadeghi Esfahlani, Louise Prothero, Naim Abdulmohdi, Naboshika Nantheswaran, Jon Turvey, Chris Jacobs

**Affiliations:** 1 Faculty of Science and Engineering, Anglia Ruskin University, Bishop Hall Lane, Chelmsford, CM1 1SQ, United Kingdom, 44 1223 695503; 2 Faculty of Health, Medicine and Social Care, Anglia Ruskin University, Chelmsford, United Kingdom; 3 SimFlow.ai Ltd, 86-90 Paul StreetLondon, United Kingdom; 4 University of Bath, Bath, England, United Kingdom

**Keywords:** autism spectrum disorder, conversational AI, digital health screening, feasibility study, acceptability, adult autism, artificial intelligence, AI

## Abstract

**Background:**

Autism spectrum disorder is underdiagnosed in adults, with increasing demand on diagnostic services and prolonged waiting times. AI-powered tools may offer scalable solutions for early screening and triage.

**Objective:**

This proof-of-concept study aimed to evaluate the feasibility, acceptability, and user experience of ASIST (Autism Screening With Intelligent Supportive Technology), an AI-powered conversational platform designed to support structured history taking and referral preparation for adults seeking to explore autistic traits.

**Methods:**

A mixed methods feasibility study was conducted. Adults (N=12) interacted with a voice-based AI chatbot delivering validated screening tools (10-item Autism Spectrum Quotient and 2-Minute Autism Detection Scale). Quantitative acceptability and usability were assessed using items informed by the theoretical framework of acceptability alongside open-text qualitative feedback.

**Results:**

Eleven patient and public involvement and engagement contributors informed the development and refinement of the study, and 12 adults completed the pilot evaluation. Participants generally reported positive perceptions of the chatbot, including low effort, favorable confidence, and perceived fairness. Open-text feedback highlighted the perceived value of ASIST as a history taking and referral support tool while also identifying areas for refinement, including pacing, speech clarity, and response format.

**Conclusions:**

AI-powered conversational tools may offer scalable solutions for structured history taking, referral preparation, and early triage. Further large-scale validation, pathway integration, and equity-focused evaluation are required before wider implementation.

## Introduction

Autism spectrum disorder is a lifelong neurodevelopmental condition affecting approximately 1% of the adult population [1]. Although awareness and early identification have improved in children, a substantial proportion of adults with autism remain undiagnosed or are diagnosed later in life [2,3]. This reflects both structural limitations in adult diagnostic services and the complexity of adult presentations, including masking behaviors, psychiatric comorbidity, and gender-related diagnostic differences [4,5].

Positive acceptability findings suggest that conversational AI may offer a practical approach to supporting preconsultation information gathering within existing referral pathways [6,7]. Many autistic adults report years of unmet needs, dismissal, or misdiagnosis prior to receiving formal recognition [8,9]. Delayed diagnosis is associated with poorer psychosocial outcomes, including anxiety and depression; reduced access to support; and difficulties obtaining accommodations [10,11]. These barriers are particularly significant for women and underrepresented groups, who may experience later diagnosis or remain unrecognized in current systems [5,12].

In addition to systemic barriers, existing adult screening and diagnostic tools also present important limitations. Many commonly used instruments were originally developed for children and later adapted for adults without sufficient adult-specific validation [13,14]. For example, the Autism Spectrum Quotient may demonstrate acceptable sensitivity but often performs poorly in terms of specificity and predictive value within adult referral populations [15,16]. In addition, co-occurring mental health conditions can confound results and further reduce diagnostic clarity [17]. More comprehensive tools such as the Autism Diagnostic Observation Schedule [18] remain resource intensive and are not designed for early-stage triage [19]. As a result, there remains a need for efficient, scalable, and adult-appropriate tools that can support earlier identification within real-world health care pathways. In response to these diagnostic and methodological challenges, AI and, more specifically, large language models (LLMs) have been proposed as a potential solution.

Machine learning approaches have demonstrated promising performance in identifying autism-related behavioral patterns from questionnaire data [20,21], whereas deep learning systems have shown potential for improving predictive accuracy in mixed-age datasets [22]. More broadly, recent advances in LLMs have expanded the capabilities of conversational AI in health care by enabling more natural, context-aware, and adaptive interactions than traditional rule-based or fixed-response systems [23]. In mental health and screening contexts, LLM-based systems may improve accessibility, scalability, and user engagement by supporting open-ended dialogue and more personalized information gathering. This is particularly relevant in adult autism assessment, where fixed-response questionnaires may not adequately capture the complexity and heterogeneity of adult presentations, especially in individuals who engage in masking behaviors or present with co-occurring psychiatric conditions [4,13,15,24]. However, important gaps remain. Existing AI studies are often based on small or nonrepresentative datasets, with limited adult-specific validation and little integration into real clinical pathways. Many systems also rely solely on structured questionnaire data and do not capture the narrative information that is often crucial in adult autism assessment. Concerns regarding transparency, bias, and fairness also remain, particularly for groups already underrepresented in autism pathways [25]. Moreover, despite progress in conversational AI, the use of LLMs in adult autism screening remains limited, with minimal integration into clinically meaningful referral workflows [11,25].

Although several digital implementations of autism screening questionnaires exist, most function as electronic versions of paper-based assessments and provide limited opportunity to capture contextual narrative information or integrate outputs directly into clinical referral workflows. Consequently, they may offer limited support for preconsultation information gathering or the structured preparation of referral documentation. These limitations provide the clinical rationale for developing conversational systems that combine validated screening measures with guided dialogue and structured reporting.

The primary contribution of this study is the design and proof-of-concept evaluation of ASIST (Autism Screening With Intelligent Supportive Technology), a workflow-based conversational AI platform designed to support structured history taking before clinical assessment. Rather than diagnosing autism or replacing clinical assessment, ASIST integrates validated screening questionnaires with guided conversational data collection to help individuals record relevant experiences and generate a structured referral summary aligned with National Institute for Health and Care Excellence (NICE) CG142 guidance. The platform is intended to complement existing clinical pathways by supporting referral preparation and reducing the administrative burden associated with information gathering during initial consultations. The aim of this proof-of-concept study was to evaluate the feasibility, acceptability, and early user experience of ASIST as a conversational platform for structured history taking and referral preparation prior to clinical autism assessment.

## Methods

### Study Design

This mixed methods feasibility study combined quantitative and qualitative approaches to evaluate the usability, acceptability, and preliminary utility of ASIST. Quantitative questionnaire data were collected following participants’ interaction with the system alongside qualitative open-text feedback. Quantitative evaluation was informed by the theoretical framework of acceptability (TFA), whereas qualitative responses were analyzed using an inductive thematic approach to identify recurring patterns in user experience and opportunities for further refinement.

The reporting of this study was informed by the CONSORT (Consolidated Standards of Reporting Trials) extension for pilot and feasibility trials where applicable to this nonrandomized feasibility study together with the World Health Organization Mobile Health Evidence Reporting and Assessment Checklist to enhance the transparency and completeness of reporting for this digital health intervention.

### AI System and Technical Configuration

ASIST was implemented using OpenAI GPT-4.1 as the underlying LLM. The conversational workflow and prompt design were informed by examples contained in the publicly available PriMock57 conversational dataset to improve dialogue quality while maintaining a structured history taking and referral workflow. The system was not designed to modify the content or scoring of the validated screening instruments but to support natural, clinically appropriate interactions throughout the assessment.

Speech-to-text transcription was performed using Deepgram, whereas text-to-speech synthesis was provided by ElevenLabs.

To ensure consistency across all participant sessions, inference parameters remained fixed throughout the study. The temperature was set to 0.7, top_p sampling was set to 0.2, and responses were limited to a maximum of 300 output tokens.

Application layer prompt engineering constrained the model to a structured clinical assessor role. Prompt instructions controlled the order of the assessment, administration of the 10-item Autism Spectrum Quotient (AQ-10) and 2-Minute Autism Detection Scale (2MADS) questionnaires, clarification of ambiguous responses, generation of the patient clinical form (PCF), and restriction of outputs to the intended structured history taking and referral support context.

The deployed system operated solely using the active conversation context and did not use internet browsing, retrieval-augmented generation, or external knowledge sources during participant interactions. Participant data were not used to train or further adapt OpenAI foundation models.

Several safeguards were implemented to promote safe and appropriate responses. Prompt-based guardrails limited the scope of the conversation to the predefined screening workflow, whereas all study sessions were monitored in real time by a medically qualified clinician. If a participant experienced distress or another safety concern arose, the clinician was available to intervene immediately. No interventions were required, and no adverse events occurred during the study.

The detailed prompt templates constitute proprietary commercial intellectual property and, therefore, cannot be reproduced in full. However, the overall system architecture, prompt control strategy, and model configuration have been described to support transparency and reproducibility.

**Figure 1.**
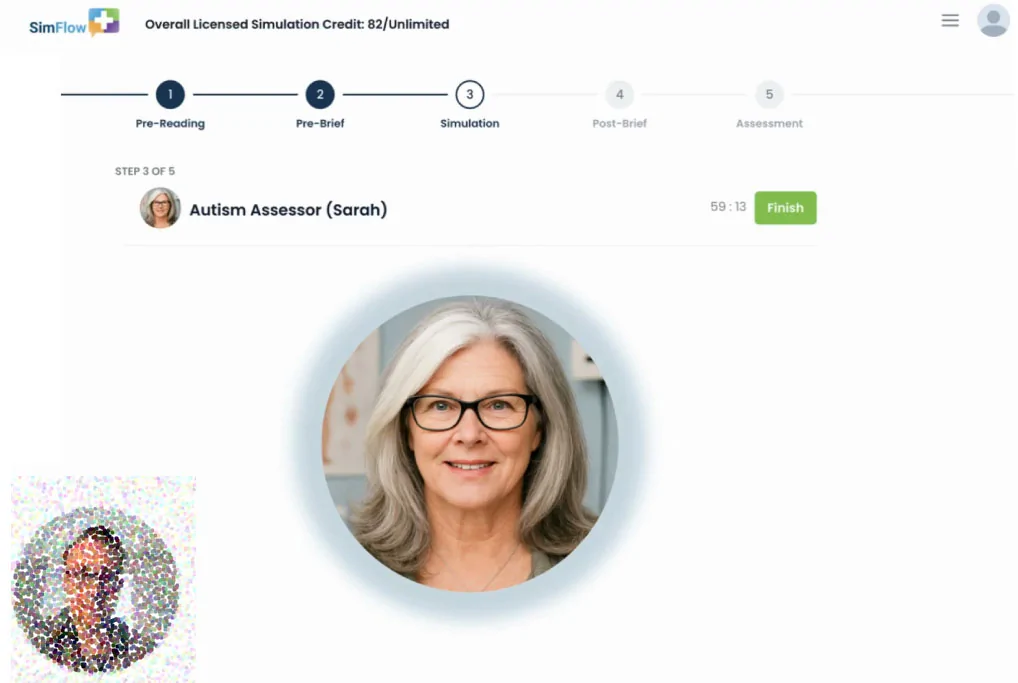
Example interface of the ASIST (Autism Screening With Intelligent Supportive Technology) conversational platform. The avatar shown was synthetically generated for demonstration purposes and was not viewed by participants during the study. Participants interacted with the system exclusively through a telephone-based voice interface. The screenshot contains no real participant information.

Figure 1 shows an example interface from the ASIST platform. The screenshot has been simplified for publication by removing nonessential interface elements. The avatar shown was synthetically generated solely for demonstration purposes and does not depict a real individual. Participants did not see this interface or avatar during the study as all interactions were conducted through a telephone-based voice interface. The screenshot contains no real participant information.

### Technical Issues During the Pilot Evaluation

During the pilot evaluation, no formal quantitative assessment of speech-to-text or text-to-speech fidelity was undertaken. Instead, conversation logs and participant interactions were reviewed by the research team to identify technical issues that could affect conversational performance. No obvious speech recognition or speech synthesis issues were identified during these reviews apart from the unintended telephony time limit described below.

During the pilot evaluation, 3 participant sessions were interrupted by an unintended 10-minute technical call limit. The affected participants completed the AQ-10 questionnaire but only part of the 2MADS assessment before the calls were terminated automatically because of the system limitation. The issue was identified during the pilot phase and resolved for all subsequent participants.

### Participants and Recruitment

Participants aged 18 years and over were recruited through convenience sampling via LinkedIn, university mailing lists, and professional networks. All participants provided informed consent before taking part. Participants represented a range of adult age groups, with most aged between 18 and 50 years (Table 1). Participants were recruited from the general adult population through convenience sampling. The study did not collect information on whether participants had a confirmed autism diagnosis, self-identified as autistic, or were awaiting clinical assessment. Consequently, the sample should be considered representative of general adult volunteers rather than the intended clinical population. Furthermore, it was not possible to determine whether participant experiences reflect those of adults most likely to use ASIST within clinical pathways. Thus, the findings should be interpreted as evidence of technical feasibility and preliminary user acceptability rather than acceptability within the target clinical population.

**Table 1.** Participant characteristics (N=12).

Characteristics	Participants
Age (y), n (%)	
18-30	4 (33.3)
31-40	3 (25)
41-50	3 (25)
51-60	2 (16.7)
Sex, n (%)	
Female	6 (50)
Male	6 (50)
Recruitment route	LinkedIn, university mailing lists, and professional networks
Interaction mode	Telephone-based voice interaction
Prior chatbot or voice assistant experience	Not collected or not reported
Autism diagnosis or self-identification	Not collected or not reported

### Ethical Considerations

#### Ethics Approval

This study received ethics approval from the Anglia Ruskin University Faculty Research Ethics Committee Panel (reference ETH2526-0428). All participants were informed about the purpose of the study, the nature of the AI-powered interaction, and the handling of audio recordings and system log data before taking part. All participants provided informed consent.

#### Security, Data Governance, and Ethics

During the evaluation, the conversational model was accessed via secure HTTPS API calls to OpenAI triggered by the telephony workflow. Accordingly, participant audio and text inputs were transmitted to the external model provider for inference. No internet browsing, external retrieval systems, or additional tools were enabled during participant sessions, and the system operated only on the active conversation context.

Audio recordings and system logs were retained solely for retrospective research analysis in accordance with the study data governance arrangements. Participants were informed about the nature of the system and provided consent before taking part. Following completion of the study, participant data processed within the SimFlow.ai platform were permanently deleted. The platform no longer retains participant data, and only the research data held by the research team are retained in accordance with institutional data governance and ethics approval.

### ASIST Platform

ASIST is an AI-powered conversational platform designed to support structured history taking before formal autism assessment. The platform is intended to help individuals organize and communicate experiences relevant to autism referral while incorporating validated screening questionnaires as one component of the information-gathering process. The system delivers a structured interaction that includes the following:

- Administration of validated screening tools (AQ-10 and 2MADS)
- Collection of structured narrative responses
- Generation of a PCF aligned with NICE CG142 guidance

The platform was deployed as a cloud-based conversational agent. All interactions were securely recorded, including audio and system logs, to enable retrospective analysis within a controlled research environment.

To enhance interaction quality, the system incorporated a sentiment-adaptive dialogue component that monitored conversational cues (eg, hesitation and clarification requests) and modulated response verbosity on a turn-by-turn basis. This enabled more natural and responsive interactions while maintaining consistency within the structured screening workflow.

### Conversational Workflow

ASIST was implemented as a structured telephony-based conversational workflow using a dedicated study phone number. Participants called the system and were guided through a predefined sequence of stages, including voice selection, consent confirmation, demographic information collection, administration of AQ-10 and 2MADS items, capture of participant explanations, score aggregation, and generation of a PCF.

**Figure 2.**
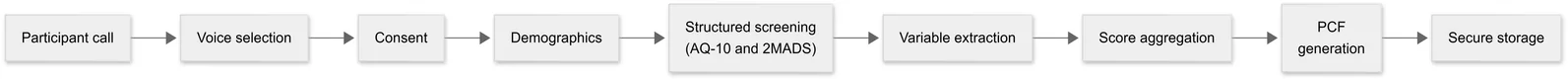
Workflow of the participant assessment and data processing pipeline. 2MADS: 2-Minute Autism Detection Scale; AQ-10: 10-item Autism Spectrum Quotient; PCF: patient clinical form.

The workflow used predefined routing logic to ensure that required screening stages were completed in a consistent order. Participant responses were captured through the telephone interface, converted from speech to text, processed by the conversational model, and returned to the participant as synthesized speech. The system was designed to support structured data capture rather than diagnostic decision-making. During the evaluation, model inference was triggered by the telephony workflow. The system did not use internet browsing, external tools, or retrieval systems and operated only on the active conversation context. To improve user interaction, the system incorporated predefined response management rules to support clarification, pacing, and conversational continuity. In cases in which participants gave incomplete or ambiguous responses, the chatbot could prompt for clarification before progressing to the next stage of the workflow. Figure 2 summarizes the deployed assessment and data processing pathway.

### Procedure

Participants completed a telephone-based ASIST session after providing informed consent. The session followed the workflow described above, including demographic questions, AQ-10 and 2MADS items, participant explanations, and generation of structured outputs for the PCF.

During each call, 2 members of the research team were present remotely via Microsoft Teams to monitor the session and provide support if required. Participants were informed that the session would be monitored for research and safety purposes. The research team did not guide responses or intervene in the chatbot interaction unless technical difficulty, uncertainty, or participant distress required support. No clinical advice or diagnostic feedback was provided during the session.

### Measures

Evaluation was conducted using bespoke questionnaire items developed specifically for this feasibility study. Questionnaire design was informed by the TFA, which provides a recognized framework for evaluating health care interventions across multiple domains of user acceptability. Because the primary aim was to assess early feasibility rather than intervention effectiveness, descriptive measures of acceptability and usability were prioritized over hypothesis testing.

Participants rated the following domains after interacting with ASIST: liking of the system, perceived effort, perceived fairness, perceived usefulness for reflection, clarity of purpose, confidence in using the chatbot, interference with other priorities, overall acceptability, and satisfaction. Items were rated on a 5-point Likert-type scale where higher scores indicated more favorable responses for liking, fairness, reflection, clarity, confidence, acceptability, and satisfaction. For effort and interference, lower scores indicated more favorable responses.

The questionnaire was designed to evaluate the following domains of the TFA:

- Affective attitude—liking of the system and overall satisfaction
- Burden—perceived effort required to use the system and interference with other priorities
- Perceived effectiveness—usefulness of the system for reflection and clarity of its purpose
- Ethicality—perceived fairness of the chatbot
- Self-efficacy—confidence in using the system

These domains were selected to provide a structured assessment of participants’ experiences with the ASIST platform. Open-text questions invited participants to describe their experience of using ASIST and suggest improvements.

### Data Analysis

Quantitative and qualitative data were analyzed separately before being interpreted together to provide an overall assessment of feasibility. Quantitative questionnaire responses were summarized descriptively using frequencies, percentages, medians, and IQRs, reflecting the ordinal nature of the data and the exploratory objectives of the study. Qualitative open-text responses were analyzed using an inductive thematic approach to identify common themes related to usability, acceptability, technical performance, and recommendations for future development.

### System Use and Completion

All participants initiated the ASIST assessment and completed the postsession usability questionnaire that formed the basis of the quantitative analyses. A total of 75% (9/12) of the participants additionally provided optional open-text feedback, which formed the qualitative dataset. No imputation of missing data was undertaken because the quantitative analyses were based solely on completed postsession usability questionnaires.

### Patient and Public Involvement and Engagement

Eleven patient and public involvement and engagement (PPIE) contributors representing a range of lived experiences—including family members of individuals with autism, professionals, and individuals with direct experience of diagnostic pathways—provided detailed feedback on the ASIST concept, design, and implementation strategy. Their responses were analyzed thematically and contributed directly to the refinement of the system and research design.

Across contributors, there was strong consensus that ASIST addresses a critical and timely gap in adult autism pathways. Participants highlighted the substantial burden associated with delayed diagnosis, long waiting times, and limited accessibility within the National Health Service (NHS). The system was widely perceived as a potentially valuable AI-powered prescreening and referral support tool that could improve access and better support individuals navigating referral pathways. Several contributors also emphasized that the platform aligned well with current health care needs and could provide meaningful support to both patients and clinicians, particularly in referral preparation and communication with primary care.

However, important concerns were raised regarding the potential overreliance on automated systems for autism screening. Contributors highlighted risks including misinterpretation or inaccurate outputs, limited personalization and nuanced understanding, absence of human empathy and 2-way interaction, and the potential emotional impact of receiving screening-related information without appropriate support. These insights reinforced the importance of positioning ASIST as a *supportive AI-powered prescreening and referral support tool* rather than a replacement for comprehensive clinical assessment, alongside the need for clear pathways to human follow-up and clinical support.

PPIE contributors also emphasized the importance of integrating ASIST within existing health care systems. Recommendations included early engagement with NHS integrated care boards, alignment with NICE guidelines and clinical workflows, consideration of training requirements for general practitioners and other professionals, and the development of a clear implementation and commissioning strategy. In addition, contributors highlighted the importance of demonstrating cost-effectiveness and clearly articulating the system’s value proposition for adoption within NHS settings.

Further feedback focused on the design of screening questions, with contributors noting the complexity and heterogeneity of autism presentations and the challenges associated with self-completing structured forms without guidance. They emphasized the need for clearer response options, explanatory support, and the inclusion of broader behavioral indicators and lived experiences. These findings supported the integration of both structured and narrative data capture within the ASIST system.

Finally, contributors highlighted the importance of ensuring that ASIST does not reinforce existing inequalities in autism diagnosis. Key recommendations included incorporating diverse populations in design and testing; accounting for gender differences, cultural context, and intersectionality; implementing bias monitoring and transparency mechanisms; and undertaking a formal equality impact assessment. The additional contributor feedback was broadly consistent with these themes, particularly reinforcing the need for coproduction, accessibility, iterative user testing, and clearer support pathways for both adults with autism and clinicians.

## Results

### Quantitative Findings

All participants (12/12, 100%) completed the postsession questionnaire; consequently, all quantitative analyses were based on 12 valid responses. A total of 75% (9/12) of participants additionally provided optional open-text feedback, which formed the qualitative dataset. Across the Likert-scale items, responses indicated generally positive perceptions of the ASIST platform. Median scores were 2 (IQR 1-2.5) for effort, 2 for interference (IQR 1-3), 3.5 (IQR 2-4) for confidence, 4 (IQR 2-4) for liking, 4 (IQR 3-4) for fairness, 4 (IQR 2.5-4) for reflection, 4 (IQR 4-4.5) for clarity, 4 (IQR 3-4) for acceptability, and 4 (IQR 3-4) for satisfaction. The clarity of the system’s purpose showed the strongest and most consistent response, suggesting that participants broadly understood how the chatbot was intended to support structured history taking and referral preparation. Perceived fairness, acceptability, satisfaction, and reflective usefulness were also rated positively (Table 2).

**Table 2.** User experience measures following interaction with ASIST (Autism Screening With Intelligent Supportive Technology).

Measures	Valid responses, n	Score (range 1-5), median (IQR)
Liking	12	4 (2-4)
Effort	12	2 (1-2.5)
Fairness	12	4 (3-4)
Reflection	12	4 (2.5-4)
Clarity	12	4 (4-4.5)
Confidence	12	3.5 (2-4)
Interference	12	2 (1-3)
Acceptability	12	4 (3-4)
Satisfaction	12	4 (3-4)

**Figure 3.**
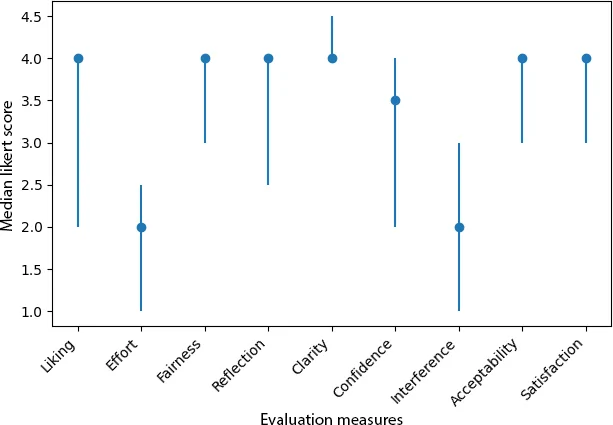
Median Likert-scale scores with IQRs across usability and acceptability measures. While most domains show positive median responses, wider IQRs for liking and confidence indicate variability in user experience.

Perceived effort was low overall (median 2, IQR 1‐2.5), indicating that most participants found the system relatively easy to use. Interference with other priorities was also low (median 2, IQR 1‐3), suggesting minimal disruption during use. However, greater variability was observed for liking of the system (median 4, IQR 2‐4) and confidence in using the chatbot (median 3.5, IQR 2‐4). This suggests that while overall usability was supported, experiences were not uniform across participants. Taken together, these results support the feasibility of ASIST as a prescreening tool while also highlighting areas where further refinement may improve consistency of user experience. Figure 3 summarizes median scores and IQRs across the user experience measures.

### Qualitative Findings

Nine participants provided open-text feedback. Four key themes were identified: interaction flow, question design, technical performance, and accessibility.

Participants reported issues with interaction flow, including delays between questions, long pauses, and abrupt call termination. These issues affected engagement and led to uncertainty about whether the system was still active. One participant described the interaction as “clunky” and similar to an “automated phone service,” whereas another noted that long pauses made them feel that the call had been disconnected.

Multipart (“stacked”) questions were frequently described as difficult to follow, with participants indicating that they were unable to respond accurately when multiple concepts were combined. Binary (“yes” or “no”) and numerical rating responses were also perceived as limiting. One participant noted that responses were “not black and white,” whereas another reported difficulty answering when “some parts applied and others didn’t.” Several participants expressed a preference for clearer response categories (eg, “never” or “sometimes”) and opportunities to elaborate on their answers.

Speech recognition and audio interaction issues were reported, including difficulties with accent recognition, pronunciation errors (particularly names), and misinterpretation of responses. Some participants experienced repeated prompts or the need to restate answers. In addition, the system’s call duration limit resulted in premature termination for several users.

Participants also highlighted the need for greater accessibility and flexibility in interaction. Several expressed a preference for multimodal input, particularly the option to use text-based responses alongside voice interaction. One participant suggested that “free text would allow more explanation,” whereas another indicated that input from family members could improve the accuracy of responses. Participants also recommended expanding the range of questions to better capture diverse autism presentations, including masking behaviors and less typical symptom profiles. Overall, the qualitative findings suggest that participants recognized the promise of ASIST as an accessible conversational platform for structured history taking and referral preparation while also identifying specific improvements needed in conversational flow, technical performance, response format, and multimodal accessibility.

## Discussion

### Principal Findings

This proof-of-concept study evaluated whether conversational AI could feasibly support structured history taking and referral preparation before formal autism assessment. The objective was not to diagnose autism or evaluate diagnostic accuracy but to determine whether adults would find a conversational approach acceptable for recording experiences relevant to subsequent clinical assessment. Overall, participants reported positive perceptions of the platform, supporting the feasibility of this approach while identifying opportunities for refinement.

Traditional paper-based autism screening questionnaires such as the AQ-10 provide an efficient method for identifying autistic traits but are inherently limited to fixed-response formats and offer little opportunity for participants to explain their experiences or place their responses in context. In contrast, ASIST combines validated screening questionnaires with conversational interaction, allowing users to elaborate on their experiences while generating structured information that may support subsequent clinical assessment.

This approach has the potential to improve the completeness and consistency of referral information while reducing administrative burden for both patients and clinicians. Importantly, autism diagnosis is a comprehensive clinical process that extends well beyond questionnaire scores. Diagnostic assessment requires detailed developmental history, clinical interview, behavioral observation, and professional judgment. ASIST was therefore not designed to determine whether an individual is autistic. Instead, it aims to support structured history taking by enabling individuals to record experiences relevant to referral before meeting a clinician. This approach has the potential to reduce the time required for information gathering during initial consultations while providing clinicians with a more organized summary of patient-reported experiences. Whether the information collected by ASIST correlates with subsequent clinical diagnoses should be evaluated in future prospective studies.

The qualitative findings provided important context for these results. Although the system was generally perceived as accessible and potentially valuable, participants identified several usability challenges, particularly related to conversational flow, question structure, response latency, and technical performance. Participants also expressed a preference for greater flexibility during conversations, including opportunities to elaborate on their responses and describe experiences that may not be adequately captured by fixed-response questionnaires.

These findings are consistent with existing research demonstrating the potential of AI-driven conversational systems to support early-stage screening and triage in health care. Digital conversational tools have been shown to improve accessibility for individuals who experience barriers to traditional health care pathways, including those with neurodevelopmental conditions. Within the NHS, prolonged waiting times for adult autism assessment remain a significant challenge, and several participants explicitly referred to delays in obtaining assessment. Although ASIST is not intended to replace validated screening instruments or comprehensive clinical assessment, it may support earlier engagement with services, standardized information collection, and more structured referral preparation.

Consistent with emerging literature, participants valued the structured nature of the assessment while expressing a preference for more adaptive and natural conversational interaction. This supports growing evidence that hybrid approaches combining validated screening instruments with conversational dialogue may improve both usability and the richness of the information collected. Previous research has similarly demonstrated that fixed-response questionnaires may inadequately capture the heterogeneity of adult autism presentations, particularly in individuals who camouflage autistic traits or present with co-occurring psychiatric conditions [4,13,15].

Several design considerations emerged from this pilot evaluation. Conversational flow should be further refined to reduce latency and avoid interruptions, whereas question design should prioritize clarity and minimize complex multipart questions that may reduce response accuracy. Participants also highlighted the importance of greater personalization, including adaptive questioning, opportunities to elaborate on responses, and alternative interaction modalities such as text input. Wider IQRs for measures of enjoyment and confidence further suggest variability in user experience, reinforcing the need for continued refinement of interaction design to accommodate the diverse communication preferences of adults with autism.

PPIE played an important role in refining both the ASIST platform and the study design. Feedback strengthened the project’s co-design approach; highlighted the importance of multimodal interaction; reinforced the need to position ASIST as a prescreening and referral support tool rather than a diagnostic substitute; and emphasized the importance of clear pathways to human follow-up, inclusive design, and bias mitigation. These contributions directly informed refinements to both the platform and the presentation of its intended clinical role.

Future evaluation should include well-characterized participant groups comprising adults with confirmed autism spectrum disorder diagnoses, individuals awaiting diagnostic assessment, those who self-identify as autistic, and neurotypical controls. This will enable robust evaluation of screening performance, user experience across different populations, and the potential role of ASIST within routine autism referral pathways.

### Clinical and Implementation Implications

Building on the proof-of-concept findings, ASIST has the potential to support referral preparation within existing NHS pathways.

In a future NHS pathway, it could support adults before or during general practitioner consultation by helping structure information relevant to referral. The generated PCF may support referral preparation by organizing information in a format aligned with NICE CG142 guidance. However, this study did not evaluate diagnostic accuracy, clinical decision-making, referral quality, waiting times, or pathway-level outcomes.

Further work is required before any claims can be made about clinical effectiveness or system-level impact. Future studies should evaluate ASIST in larger and more diverse samples; compare outputs against established clinical assessment pathways; and examine acceptability among clinicians, patients, and commissioners.

LLM-based systems also introduce risks related to response variability, inaccurate interpretation, hallucination, and hidden bias. Although ASIST mitigates some of these risks through organized workflow design, constrained prompts, and validated screening instruments, further validation is required to establish reliability, equity, clinical safety, and pathway-level utility.

### Limitations

This study has several limitations that should be considered when interpreting the findings.

First, participants’ autism diagnostic status was not collected. Consequently, it is unknown whether the sample comprised formally diagnosed adults with autism, individuals awaiting assessment, those who self-identified as autistic, or individuals simply exploring autistic traits. Therefore, the findings should be interpreted as reflecting the acceptability and usability of ASIST among adults interested in exploring autistic traits rather than among any specific clinical population. Future studies should record participants’ diagnostic status and recruitment pathway to better characterize the target population and examine whether acceptability differs across these groups.

Second, the evaluation involved a small convenience sample of 12 participants, limiting the generalizability of the results. Participants were recruited through convenience sampling and may not be representative of the wider adult population seeking autism assessment. Consequently, the findings may be influenced by selection bias, and user experiences observed in this study may differ in more diverse clinical and community populations.

Third, the primary objective of this study was to evaluate the feasibility and acceptability of ASIST as a proof-of-concept conversational platform for structured history taking and referral preparation rather than its diagnostic accuracy or clinical effectiveness. The study was not powered to evaluate diagnostic performance, validate screening accuracy, or compare ASIST with existing screening approaches. Therefore, the findings should be interpreted as preliminary evidence supporting the feasibility and user acceptability of the platform.

Finally, the study evaluated ASIST within a controlled pilot setting. Further research involving larger, more representative populations, multiple clinical sites, and direct comparisons with existing screening pathways is required to establish the platform’s reliability, clinical utility, and integration into routine health care practice.

### Conclusions

ASIST is a voice-based conversational platform designed to support structured history taking and referral preparation before formal autism assessment. This proof-of-concept study demonstrates that conversational AI can feasibly support the collection of structured patient-reported information while incorporating validated screening questionnaires within an accessible dialogue. Future work should determine whether information generated by ASIST improves referral quality and correlates with subsequent clinical diagnosis.

## Abbreviations

2MADS: 2-Minute Autism Detection Scale
AQ-10: 10-item Autism Spectrum Quotient
ASIST: Autism Screening With Intelligent Supportive Technology
CONSORT: Consolidated Standards of Reporting Trials
LLM: large language model
NHS: National Health Service
NICE: National Institute for Health and Care Excellence
PCF: patient clinical form
PPIE: patient and public involvement and engagement
TFA: theoretical framework of acceptability
